# Clinical features and risk factors of Raynaud’s phenomenon in primary Sjögren’s syndrome

**DOI:** 10.1007/s10067-021-05749-w

**Published:** 2021-04-29

**Authors:** Wei Lin, Zhifei Xin, Xiaoran Ning, Yang Li, Xiuying Ren, Yashuang Su, Meilu Liu, Shaoying Guo, Liu Yang, Yixuan Liu, Fengxiao Zhang, Wen Zhang

**Affiliations:** 1 Department of Rheumatology and Immunology, Hebei General Hospital, No. 348 Heping West Road, Shijiazhuang, 050051 Hebei China; 2 Department of Thoracic Surgery, Hebei General Hospital, Shijiazhuang, 050051 China; 3 Department of Oncology, Hebei General Hospital, Shijiazhuang, 050051 China; 4 Department of Graduate School, Hebei Medical University, Shijiazhuang, 050017 China; 5 Department of Rheumatology, Peking Union Medical College Hospital, Chinese Academy of Medical Science & Peking Union Medical College, National Clinical Research Center for Dermatologic and Immunologic Diseases, State Key Laboratory of Complex Severe and Rare Diseases, Beijing, 100730 China

**Keywords:** Interstitial lung disease, Primary Sjögren’s syndrome, Raynaud’s phenomenon, Risk factors

## Abstract

**Objective:**

The aim at the current study was to investigate the clinical characteristics and risk factors of Raynaud’s phenomenon (RP) in patients with primary Sjögren’s syndrome (pSS).

**Methods:**

Retrospective analysis of the medical records of 333 new-onset pSS patients was performed. Demographic, clinical, and serological data were compared between individuals with and without RP. Logistic regression analysis was used to identify risk factors.

**Results:**

RP was present in 11.41% of the pSS patients. pSS-RP patients were younger (49.74±14.56 years vs. 54.46±13.20 years, *p*=0.04) and exhibited higher disease activity (11 [5.75–15] vs. 7 [4–12], *p*=0.03) than those without. The prevalence of lung involvement was significantly higher in pSS patients with RP (60.53% vs. 17.29%; *p*<0.001). A significantly higher proportion of patients with pSS-RP tested positive about antinuclear (ANA), anti-RNP, and anti-centromere antibodies (ACA) compared to those without (*p*=0.003, <0.001, and 0.01, respectively). Multivariate analysis identified lung involvement (odds ratio [OR]=8.81, 95% confidence interval [CI] 2.02–38.47; *p*=0.04), anti-RNP positive status (OR=79.41, 95% CI 12.57–501.78; *p*<0.0001), as well as ACA (OR=13.17, 95% CI 2.60–66.72; *p*=0.002) as prognostic factors for pSS-RP.

**Conclusion:**

The presence of RP defined a subset of pSS with a unique phenotype, manifesting as increased lung involvement and a higher frequency of anti-RNP antibodies and ACA, as well as greater disease activity. These results suggest that RP has clinical and prognostic value of pSS patients. Further prospective studies with a larger number of subjects are warranted to confirm our findings and assess the prognostic and treatment implications of RP in pSS patients.

**Table:** 

**Key Points** • *Raynaud’s phenomenon (RP) was present in 38 (11.41%) of 333 patients with primary Sjögren’s syndrome (pSS), with patients with RP exhibiting a younger age and higher disease activity.* • *The presence of RP indicates a subset of pSS with a unique phenotype, with manifestations including increased lung involvement and a higher frequency of anti-RNP antibodies and anti-centromere antibodies.* • *Patients with pSS and RP need close follow-up and long-term observation (including assessment of microangiopathy), with specific attention paid to the possible development of clinical features of systemic sclerosis.*

**Supplementary Information:**

The online version contains supplementary material available at 10.1007/s10067-021-05749-w.

## Introduction

Primary Sjögren’s syndrome (pSS) is a chronic systemic autoimmune disease characterized by lymphocytic infiltration of the exocrine glands and circulating B-cell hyperactivity, with a wide range of organ-specific and systemic manifestation [1–5]. Dryness of the mouth and eyes is the most prevalent symptoms of the syndrome, while extraglandular disorders (including inflammatory arthritis, cutaneous lesions, and interstitial lung diseases) occur to approximately one-third of patients [6].

Raynaud’s phenomenon (RP) is essentially an exaggerated vasospastic response to cold exposure or stress, with the classic triphasic color change progressing from white (ischemia), from blue (de-oxygenation) to red (reperfusion) [7, 8]. Primary or secondary to several different conditions or causes, RP is often the initial manifestation of an underlying autoimmune connective tissue disease, such as systemic sclerosis (SSc), systemic lupus erythematosus (SLE), mixed connective tissue disease (MCTD), or Sjögren’s syndrome (SS) [9, 10]. Previous studies evaluating the prevalence of RP in patients with pSS have reported conflicting results with the prevalence ranges from 9 to 33% [11–14]. Additionally, some studies have linked the presence of RP to special clinical manifestations, including pulmonary hypertension (PH) and interstitial lung disease (ILD) [15].

Currently, it remains unknown whether the presence of RP represents a distinct subtype of the pSS. To the best of our knowledge, there are no reports describing the clinical features of comorbid pSS-RP in Chinese patients. We performed a retrospective study to investigate the prevalence, clinical characteristics, and immunological features of RP in patients with pSS.

## Materials and methods

### Study design and data collection

A total of 333 patients that received a new diagnosis of pSS between January 2016 and March 2019 in the inpatient Department of Rheumatology and Immunology of the Hebei General Hospital were retrospectively reviewed. The diagnosis of pSS was based on the 2002 American-European Consensus Group criteria [16]. Patients were divided into two groups according to the presence (pSS-RP cases) or absence (pSS controls) of RP. This study was approved by the Clinical Research Ethics Committee of the Hebei General Hospital (NO.2016070), and written informed consent for publication was obtained from the patients.

### Data collection

Data regarding demographic, clinical, and serological profiles were retrospectively collected from medical records at the time of initial pSS diagnosis. The clinical information included age, disease duration, and systemic complications such as joint impairment, skin involvement, digestive involvement, renal dysfunction, neurological manifestations, pulmonary involvement, and hematological impairment [17, 18]. Laboratory findings included complete blood counts, erythrocyte sedimentation rate (ESR), C-reactive protein (CRP), serum immunoglobulins (Ig), complement 3 (C3), complement 4 (C4), rheumatoid factor (RF), and autoantibodies. Autoantibodies included ANA, anti-Sm, anti-RNP, anti-Ro/SSA, anti-La/SSB, and ACA. The samples of all tests were fresh blood, which were collected at first visit to our hospital. Additionally, the European League Against Rheumatism Sjögren’s Syndrome Disease Activity Index (ESSDAI) was used to evaluate disease activity of all pSS patients [19]. All data are available.

### Statistical analysis

SPSS for Windows 25.0 statistical software package (IBM Corp., Armonk, NY, USA) was used for statistical analysis. Continuous variables were expressed as means and corresponding standard deviations for normally distributed data, with medians with 25–75th percentiles (Q25–Q75) used for presenting non-normally distributed data. Student’s *t*-test, chi-squared tests, and the Mann-Whitney *U* test were used for comparisons. Logistic regression analysis was performed to identify risk factors associated with RP in patients with pSS. We performed the analysis in two stages. First, we performed a monofactor analysis to examine differences between pSS patients with and without RP. Next, variables with *p* values ≤0.1 were entered into a multiple logistic regression analysis to provide adjusted estimations of the odds ratio (OR). OR with 95% confidence interval (95% CI) was calculated within the logistic regression analysis. In all statistical analyses, *p* < 0.05 was considered statistically significant.

## Results

A total of 333 new-onset pSS patients were analyzed. In this series, 93.09% of patients were females, while 6.91% were males. Among the patients, 38 patients with pSS were complicated with RP, with a prevalence of 11.41%.

### Demographic characteristics and immunological features of pSS patients with and without RP

Comparing pSS patients with and without RP, patients with RP were diagnosed at an earlier age (49.74±14.56 years) than patients without RP (54.46±13.20 years; *p*= 0.04). No significant differences were found between the two groups in percentages of white blood cells as well as the subsets. While the circulating levels of RF and IgG were higher in patients with pSS and RP compared to patients without RP, the difference was not statistically significant. A significantly greater number of patients with pSS and RP were positive for ANA, ACA, and anti-RNP antibodies (*p* = 0.003, 0.01, and <0.001, respectively), while no significant differences were observed in other antibodies, such as anti-SSA, anti-SSB, and anti-Ro 52 (Table 1).

**Table 1:** Baseline characteristics of pSS patients with and without RP

	pSS with RP (*n*=38)	pSS without RP (*n*=295)	Unadjusted OR	95% CI	*p* value
Demographic features					
Gender (F/M)	37:1	273:22	0.34	0.04–2.56	0.49
Age (years)	49.74±14.56	54.46±13.20	-	-	0.04
Duration (months)	60 [24–120]	48 [12–120]	-	-	0.18
Laboratory findings					
White blood cell counts (×10^9^/L)	4.62 [3.58–6.23]	4.97 [3.97–6.29]	-	-	0.37
Neutrophil counts (×10^9^/L)	2.84 [1.79–4.37]	2.94 [2.21–4.25]	-	-	0.36
Lymphocyte counts (×10^9^/L)	1.42 [1.02–1.79]	1.52 [1.10–1.87]	-	-	0.54
Hemoglobin (×g/L)	118 [109–133.75]	122 [110–132]	-	-	0.65
Platelet counts (×10^9^/L)	200 [165.25–263.75]	227 [179–270]	-	-	0.31
NLR	1.78 [1.19–3.10]	2.08 [1.43–2.91]	-	-	0.47
PLR	148.42 [98.64–198.74]	146.88 [110.56–206.92]	-	-	0.75
ESR (mm/1 h)	18 [11.5–33]	19 [8.25–36]	-	-	0.98
CRP (mg/L)	2.67 [0.9–5.82]	3.3 [1.12–4.29]	-	-	0.83
RF (IU/L)	25.5 [10.60–67]	16.2 [10.60–68.85]	-	-	0.71
IgG (g/L)	16.50 [13.89–21.41]	15.16 [12.10–19.65]	-	-	0.13
IgA (g/L)	2.85 [1.99–3.72]	2.06 [1.87–3.09]	-	-	0.02
IgM (g/L)	1.17 [0.82–1.62]	1.12 [0.74–1.60]	-	-	0.51
Elevated immunoglobulins (*n*, %)	14/38, 36.84%	103/289, 35.64%	1.04	0.51–2.09	0.88
RF (+) (*n*, %)^a^	20/35, 57.14%	130/276, 47.10%	1.50	0.74–3.05	0.26
ANA (+) (*n*, %)^b^	37/97.37%	226/76.61%	11.30	1.52–83.85	0.003
Anti-RNP (+) (*n*, %)	24/63.16%	15/5.08%	32.00	13.83–74.07	<0.001
Anti-Ro52 (+)	24/63.16%	176/59.66%	1.16	0.58–2.33	0.68
Anti-Ro/SSA (+) (*n*, %)	19/50%	168/56.95%	0.76	0.38–1.49	0.42
Anti-La/SSB(+) (*n*, %)	6/15.79%	73/24.75%	0.57	0.23–1.42	0.22
ACA (+) (*n*, %)	10/26.32%	35/11.86%	2.65	1.19–5.93	0.01
Pathological of MSG with focus score ≥1 (*n*, %)	36/37, 97.30%	273/286, 95.45	1.71	0.22–13.50	1.0

*pSS*, primary Sjögren’s syndrome; *RF*, rheumatoid factor; *ANA*, antinuclear antibodies; *MSG*, minor salivary gland; *ACA*, anti-centromere antibodies; *RNP*, ribonucleoprotein. ^a^Positive RF >20 IU/mL; ^b^positive for ANA titers ≥1:320

### Clinical characteristics of pSS patients with RP

A comparison of clinical characteristics between pSS patients with and without RP is shown in Table 2. Significant differences were found for lung involvement (*p*< 0.001), including ILD (*p* < 0.001) and PAH (*p* = 0.03), between the two subgroups. Additionally, leukopenia and lymphopenia were found more frequently in pSS patients with RP than in patients without RP (leukopenia: 31.58% vs. 17.63%, respectively, *p*=0.04; lymphopenia: 34.21% vs. 25.42%, respectively, *p*=0.25). No significant differences were observed in renal, digestive, and nervous system involvement between the groups (*p* > 0.05). Further, pSS patients with RP exhibited higher pSS disease activity (*p* = 0.03; Table 2).

**Table 2:** Clinical features between pSS patients with or without RP

Variables	pSS with RP (*n*=38)	pSS without RP (*n*=295)	Unadjusted OR	95% CI	*p* value
Xerostomia (*n*, %)	33 (86.84%)	257 (87.12%)	0.98	0.36–2.65	1.0
Xerophthalmia (*n*, %)	30 (78.95%)	216 (73.22%)	1.37	0.60–3.12	0.45
Salivary gland enlargement (*n*, %)	6 (15.79%)	32 (10.85%)	1.54	0.60–3.97	0.41
Systemic involvements (*n*, %)					
Hematological involvement	26 (68.42%)	159 (53.90%)	1.85	0.90–3.81	0.09
Thrombocytopenia	3 (7.89%)	17 (5.76%)	1.40	0.39–5.03	0.49
Leukopenia	12 (31.58%)	52 (17.63%)	2.16	1.02–4.55	0.04
Lymphopenia	13 (34.21%)	75 (25.42%)	1.53	0.74–3.13	0.25
Hypocomplementemia	10 (26.32%)	75 (25.42 %)	1.07	0.50–2.30	0.91
Arthritis	20 (52.63%)	126 (42.71%)	1.49	0.76–2.93	0.25
Lung involvement	23 (60.53%)	51 (17.29%)	7.34	3.58–15.03	<0.001
Interstitial lung disease	22 (57.89%)	44 (14.92%)	7.84	3.82–16.10	<0.001
Pulmonary hypertension	6 (15.79%)	16 (5.42%)	3.27	1.19–8.95	0.03
Renal involvement	1 (2.63%)	21 (7.12%)	0.35	0.05–2.70	0.49
Digestive involvement	2 (5.26%)	15 (5.08%)	1.04	0.23–4.72	1.0
Nervous system involvement	2 (5.26%)	39 (13.22%)	0.37	0.08–1.58	0.20
Mucocutaneous involvement	38 (100%)	46 (15.59%)	1.81	1.49–2.19	<0.001
Lymphatic system involvement	7 (18.42%)	26 (8.81%)	2.34	0.94–2.19	0.08
ESSDAI	11 [5.75–15]	7 [4–12]	-	-	0.03

### Potential risk factors for RP development in patients with pSS

Multivariate logistic regression analysis was performed to identify the risk factors that can predict the development of RP in pSS patients (Table 3). Pulmonary and mucocutaneous involvement, ANA, anti-RNP antibodies, ACA, and variables found to be significantly different between pSS patients with and without RP were included in the assessment. Lung involvement (OR=8.81, 95% CI 2.02–38.47; *p*=0.004), the presence of circulating anti-RNP antibodies (OR=79.41, 95% CI 12.57–501.78; *p*<0.001), and ACA (OR=13.17, 95% CI 2.60–66.72; *p*=0.002) were found to be positively associated with RP in patients with pSS (Table 3). Other factors were not significantly related to the development of RP in pSS patients.

**Table 3:** Multivariate analysis of features predicting RP in pSS patients

	*p* value	Adjusted OR	95% CI
ANA positive	0.23	4.54	0.39–52.85
Anti-RNP positive	<0.001	79.41	12.57–501.78
ACA positive	0.002	13.17	2.60–66.72
Pulmonary involvement	0.004	8.81	2.02–38.47

*CI*, confidence interval; *OR*, odds ratio

## Discussion

In this study, for the first time, we investigated the differences of the specific clinical and immunological characteristics between pSS patients with and without RP, and evaluated the potential risk factors for comorbid pSS-RP in Chinese populations. RP was more common to people that experience disease onset of a younger age and in patients with severer pSS, including manifestations such as higher prevalence of lung damage (including ILD and PH). Additionally, this subgroup of patients with pSS is serologically marked by the presence of anti-RNP antibodies and ACA. Further, the involvement in lung tissue may be a risk factor of the development of RP.

With regard to the prevalence of RP in pSS patients, Youinou P et al. [12] previously described as 33.33% out of 45 pSS patients in France. Prevalence in our data (11.41%) was statistically lower than that of their data (*p*<0.001 by chi-square test), but was comparable to the data from Spain reported by Garcia-Carrasco M et al. (12.5%, *p*=0.67 by chi-square test) [14] *and* the data from Argentina reported by Demarchi J et al. (9.05%, *p*=0.37 by chi-square test) [20]. Several factors account for the wide range of prevalence (data summarized in sup-Table 1) estimates of the literature. Notably, genetic has been indicated in the pathogenesis of RP. In 2000, Susol E. et al. conducted a genome-wide screen to identify chromosomal regions containing genes conferring susceptibility to RP, and identified three potential candidate genes: the beta subunit of the muscle acetylcholine receptor and the serotonin 1B and 1E receptors by a two-stage screening process [21]. Crispin JC et al. assessed the association between RP and single nucleotide polymorphisms (SNPs) in temperature-responsive or vasospastic genes and found that RP was associated with variation in gene NOS1 [22]. Additionally, it has been suggested that MTHFR mutation is more frequently found in patients with RP, which confirms genetic predisposition may play a key role in the pathogenesis of RP [23].What’s more, geographical factors may also lead to differences, because a colder climate may promote the development of RP [24]. Further, 37 of our 38 patients with pSS and RP were women, consistent with the results of other studies, showed that the incidence of RP in women was higher, suggesting that hormonal factors played an important role in the expression of this disorder. And some studies have found an association for RP and use of estrogen replacement therapy alone [25, 26].

A key finding in our study comes from the comparison of pSS disease activity between patients with and without RP. In the present study, we found the ESSDAI in patients with pSS and RP was significantly higher than in patients without RP. This finding contrasted to the report on Heimovski et al., who found lower disease activity score in SLE patients with RP [27]. In our current study, pSS-RP patients had more frequent extraglandular features compared to patients without RP, specifically lung involvement and cutaneous vasculitis. This observation is in line with previous published reports. Garcia-Carrasco et al. reported a higher prevalence of articular involvement and cutaneous vasculitis [14] in pSS patients with RP, while Kraus et al. observed a higher frequency of non-erosive arthritis, vasculitis, and pulmonary fibrosis in patients with both pSS and RP [13]. Additionally, Roca et al. suggested that pSS patients with ILD more commonly exhibited RP [28]. Furthermore, RP was found to be associated with sensorimotor neuropathy and mononeuritis multiplex [29]. These reports show a similar pattern of extraglandular features, suggesting that pSS patients with RP comprise a subset with a homogeneous clinical presentation. Genetic predisposition was proposed as a potential contributor to the RP onset of pSS. Specifically, a subset of studies reported an increased frequency of HLA-DRw3 and HLA-DRw4 alleles [30] in patients with RP and pSS, which could partly account for the differences observed between pSS patients with and without RP.

Our results indicate that lung involvement may be a risk factor for the development of RP. Lung disorders, such as ILD, are common complications associated with autoimmune diseases, with very limited effective treatment strategies currently available [31]. In recent years, pSS patients with ILD were reported in some studies to exhibit RP more commonly [28], suggesting that an ischemic process may play a role in the development of lung damage. Furthermore, the results of our current study confirm the relationship between ILD and RP in pSS patients, expanding our understanding of the pathological mechanisms in ILD. Our findings indicate that assessment of microangiopathy should become an integral part of clinical evaluation of patients with pSS to allow the early detection of ILD or other fibrosis damage.

In addition, RP is associated with immunological disturbances. Previous published studies on immunological characteristics have provided conflicting information. Garcia-Carrasco et al. reported a higher frequency of immunological markers in patients with SS and RP, primarily ANA and anti-Ro/SSA [14]. Conversely, other studies found no differences in immunological measures [11–13]. In our study, we noted the previously established association of RP with ANA, ACA, and anti-RNP autoantibodies, which were in agreement with two recent reports [32, 33]. A growing number of publications described the subgroup of pSS which displays a distinct serological profile characterized by the presence of ACA, which is well known to appear in CREST syndrome or limited cutaneous SSc patients [34]. ACA-positive pSS patients are more frequently affected by RP, peripheral neuropathy, and sclerodactyly, as compared to ACA-negative patients with pSS. Additionally, based on previous studies, pSS patients with positive ACA were regarded as a distinct clinical subgroup, showing intermediate features on classical pSS and limited cutaneous scleroderma (lSSc) [34]. Furthermore, Miyawaki et al. [35] reported that 6 out of 10 (60%) pSS patients with ACA and RP developed CREST syndrome in the long follow-up investigation, and it is necessary for us to follow up the present ACA-positive pSS-RP patients to determine whether they develop CREST syndrome in the long run. Although we excluded SSc in the present study according to the criteria [16], pSS complicated with SSc should be carefully examined during follow-up period. A prospective study for ACA-positive pSS-RP subset, with closer follow-up and more long-term observation, is needed to make mention of possibility for development to CREST syndrome or SSc.

There are several limitations on this study. We must first underline the size difference between the two groups. Even if we included 333 pSS patients, the largest number of cohort patients compared with the previous studies for pSS-RP, we had only 38 patients with RP. The relatively small size of RP patients might have lacked statistical power to show some differences and made it difficult to draw definite conclusions. Second, this is a retrospective, single-center study; the lack of clinical information on some patients may lead to unexpected various biases. Further prospective studies with a larger number of subjects are warranted to confirm our findings and assess the prognostic and treatment implications of RP in pSS patients.

In summary, our current findings confirm that observation of RP, despite being considered a non-specific cutaneous lesion, provides important information on the distinct systemic and immunological profiles of this subcategory of patients with pSS. The detection of RP allows for the establishment of a disease subgroup in which patients experience pSS with an onset of a younger age and a more severe clinical presentation, including manifestations such as higher prevalence of lung damage (including ILD and PH). Additionally, this subgroup of patients with pSS is serologically marked by the presence of anti-RNP and ACA. Furthermore, the involvement of lung tissue may be a risk factor of the development of RP, which suggests a number of potential treatment targets. Further large-scale prospective studies are warranted to confirm these findings.

## Supplementary Information

ESM 1 (DOCX 15 kb).

## Data Availability

The datasets used or analyzed during the current study are available from the corresponding author on reasonable request.
