# Supplementary material for: Clinical features and risk factors of Raynaud’s phenomenon in primary Sjögren’s syndrome
Source: Clin Rheumatol. 2021 Apr 29;40(10):4081–7. doi: 10.1007/s10067-021-05749-w (PMC8463379; doi:10.1007/s10067-021-05749-w)
Supplement: Supplementary file 1 — (DOCX 15 kb). [file 10067_2021_5749_MOESM1_ESM.docx]

***Sup-Table 1.* previous studies of RP in patients with pSS**

|  | Skopouli 1990 | Youinou 1990 | Kraus 1992 | Garcia-Carrasco, 2002 | Present Study, 2021 |
| --- | --- | --- | --- | --- | --- |
| Number of pSS patients | 110 | 45 | 104 | 320 | 333 |
| Country | Greece | France | Mexico | Spain | China |
| pSS criteria | Greek | Greek | Fox | European | American-European |
| Patients with RP (n, %) | 36, 32.73% | 15, 33.33% | 30, 28.85% | 40, 12.5% | 38, 11.41% |
| Females (n, %) | ND | ND | ND | 40, 100% | 37, 97.37% |
| Clinical associations | Swollen hands | Arthritis | Arthritis, | Arthritis, | Lung involvement, |
|  |  |  | vasculitis, | cutaneous vasculitis | mucocutaneous involvement |
|  |  |  | pulmonary fibrosis, |  |  |
| Immunological associations | NS | NS | NS | ANA, A-Ro/SSA, A-La/SSB | ANA, A-RNP, ACA |
| ND: no data. NS, not significant. |  |  |  |  |  |
